# The effect of a mindfulness-based cognitive therapy program on the spiritual health of mothers of infants admitted to the neonatal intensive care unit

**DOI:** 10.3389/fpsyg.2024.1346661

**Published:** 2025-01-07

**Authors:** Naeime Arasteh, Reihane Moghimian Shahrbabaki, Monirsadat Nematollahi, Atefe Ahmadi, Behnaz Bagherian

**Affiliations:** ^1^Nursing Research Center, Kerman University of Medical Sciences, Kerman, Iran; ^2^Reproductive and Family Health Research Center, Kerman University of Medical Sciences, Kerman, Iran

**Keywords:** cognitive therapy, mindfulness, spiritual health, mothers, infants, neonatal intensive care unit

## Abstract

**Introduction:**

The birth and admission of a premature infant to the NICU is often an unexpected experience and a mental and spiritual challenge for families. Spiritual health is an influential factor affecting how a mother faces and endures a stressful situation. Improving the mother's spiritual health requires cognitive therapy approaches, including mindfulness techniques. Nurses can help mothers of infants admitted to the NICU to go through the hospitalization period more peacefully by using mindfulness techniques. To this end, the present study sought to investigate the effect of a mindfulness-based cognitive therapy (MBCT) program on spiritual health in mothers of infants admitted to the NICU.

**Methods:**

This quasi-experimental study was conducted using a pre-test-post-test design in two intervention and control groups. The participants were 50 mothers of infants admitted to the NICU of Afzalipur Hospital, Kerman, who were selected via convenience sampling and randomly divided into two control and intervention groups (25 mothers in each group). The intervention involved providing counseling using a short-term mindfulness-based cognitive therapy program carried out in four 2-h sessions for the participants in the intervention group. The participants in the control group received routine NICU care. The participants in both groups completed a demographic information questionnaire and the Spiritual WellBeing Scale (SWBS) before and after the intervention. The collected data were analyzed with SPSS-25 software.

**Results:**

The data showed no statistically significant difference between the two groups in terms of demographic characteristics (*P* > 0.05). Moreover, the mean score of spiritual health in the intervention group was 92.80 ± 12.14 and that of the control group was 92.16 ± 16.35 before the intervention. The mean score of spiritual health in the intervention group was 104.04 ± 6.60 and that of the control group was 92.56 ± 16.35 after the intervention. The results of the paired samples *t*-test revealed a statistically significant difference in the mean scores of spiritual health and its dimensions before and after the intervention in the intervention group (*P* < 0.001).

**Discussion:**

The findings from the present study indicated that mindfulness-based cognitive therapy intervention was effective in improving the spiritual health of mothers with infants admitted to the NICU. NICU nurses can use mindfulness-based cognitive therapy as a practical intervention to improve the spiritual health of mothers of premature infants and promote the sense of meaningfulness and meaning of life in these mothers.

## Introduction

Infants are admitted to the neonatal intensive care unit (NICU) for various reasons including prematurity, congenital anomalies, sepsis, and heart, gastrointestinal, or neurological abnormalities. Admission to the NICU is a stressful experience for the mother and the infant and can be associated with a feeling of helplessness and psychological distress in the mother. Moreover, the mother's worries about the infant's survival and the inability to communicate with and care for the infant lead to the loss of self-control, fear, and anxiety in the mother (Mendelson et al., 2018; Gerstein et al., 2019; Rihan et al., 2021; Grieb et al., 2023).

During the infant's stay in NICU, mothers are exposed to many stressors including unknown medical condition of the infant, limited access to the infant due to monitor leads, feeding and breathing tubes, and the incubator leading to restlessness in the mother. Moreover, limited information about the treatment process makes it more difficult for the mother to endure this critical period. All these stressors have permanent negative effects on the mother's mental health (Gerstein et al., 2019; Grieb et al., 2023).

Thus, one of the main functions of nurses is to provide emotional support to these mothers and pay attention to their stress and distress to improve the mother's health and wellbeing. Many studies have shown higher levels of psychological distress in mothers of infants admitted to the NICU (Gerstein et al., 2019; Maleki et al., 2022; Malmir et al., 2022). However, less attention has been paid to the mother's spiritual health (Grieb et al., 2023).

In particular, the NICU is a stressful and critical environment (Sadeghi et al., 2016). Infants' admission to the NICU is a sad and traumatic experience for the mother. Besides, the challenges experienced by the mother during the hospitalization of her child inevitably lead to a spiritual crisis in the mother. Thus, the mothers ask questions about the meaning of life and health, and their spirituality is also affected (Sadeghi et al., 2016).

Spiritual health has two horizontal and vertical dimensions and refers to a feeling of connection with others and understanding the meaning of life (existential health) and a connection with the supernatural force (religious health) that helps people live better and interact more effectively with the world around them (Dehghan et al., 2021; Malmir et al., 2022).

Moreover, spiritual health is associated with other (physical, mental, and social) aspects of health and affects individuals' mental and physical health (François et al., 2019). Numerous studies have shown that spiritual health disorders or spiritual distress cause mental disturbances, anxiety, depression, and loss of the meaning of life (Asadzandi, 2017; Malmir et al., 2022). In contrast, the promotion of spiritual health can lead to the reduction of distress, anxiety, and stress, increased adaptability, and higher levels of coping skills and resilience in mothers with stressful experiences in the NICU, hence increasing the mother's desire to communicate with her infant and attachment to the infant and mother's wellbeing (Mollica et al., 2016; Sankhe et al., 2017; Bockrath et al., 2022; Malmir et al., 2022). Thus, nurses should evaluate the spiritual health of mothers to reduce the possibility of deeper spiritual distress (Sadeghi et al., 2016). A study showed that nurses pay attention to the significance of the religious and spiritual concerns of families and consider it their duty to respond to the spiritual needs of patients and their families (Robinson et al., 2006). However, conducting nursing interventions such as mindfulness-based interventions for improving the spiritual health and mental peace of mothers can help respond to the needs of patients' families (Walker, 2020; Grieb et al., 2023).

Mindfulness is rooted in ancient spiritual traditions and is at least 2,550 years old and has been systematically expressed in Buddhism. Mindfulness is a state in which a person has purposeful and non-judgmental attention to the present moment. It is positively associated with factors related to health and wellbeing and inversely associated with psychological distress (Asani et al., 2023). Mindfulness is also considered a potentially effective intervention against psychological distress, anxiety, worry, fear, anger, and excessive preoccupation with distressing thoughts and feelings (Keng et al., 2011).

Grieb et al. (2023) showed that mindfulness exercises help mothers of infants admitted to the NICU establish a different relationship with the worries, uncertainties, challenges, and stress experienced in the NICU and reduce unpleasant emotional states through greater self-compassion.

According to studies, mindfulness exercises may improve metacognitive awareness and allow mothers to deal differently with emotional challenges, thereby reducing anxiety and depression, improving the ability to cope with difficult conditions experienced in the NICU, and improving wellbeing, quality of life, and pain relief, and making life more meaningful (Mendelson et al., 2018; Grieb et al., 2023; Leng et al., 2023).

Mindfulness-based cognitive therapy (MBCT) is an evidence-based group program that focuses on promoting non-judgmental awareness of the present moment through meditation and cognitive-behavioral exercises (Bredero et al., 2023). MBCT enables an individual to shift her attention from recurrent negative thoughts to the here and now, and as a result, can prevent rumination caused by negative thoughts and the occurrence of depression. The goal of MBCT is to create a non-judgmental and compassionate attitude toward negative thoughts and feelings to regulate emotions and positive mood and promote psychological wellbeing (Bhattacharya and Hofmann, 2023; Tseng et al., 2023).

Generally, providing comprehensive care and responding to the needs of patients and their families is one of the important nursing functions, and nurses can conduct MBCT interventions to relieve the intensity of psychological problems of mothers and the impact on their spiritual health. This being so, the present study aimed to investigate the effect of a short-term mindfulness-based cognitive therapy program on the spiritual health of mothers of infants admitted to the NICU.

## Methods

### Research design and setting

This quasi-experimental study was conducted on the mothers of infants admitted to the NICU of Afzalipur Hospital in Kerman. This hospital has 70 neonatal intensive care beds, which provide level-3 medical and nursing services to patients.

### Participants

The participants in this study were mothers of premature infants admitted to the NICU of Afzalipur Hospital in Kerman in 2022. The participants were selected through convenience sampling and divided into two intervention and control groups randomly using a random number table. The inclusion criteria were having infants admitted to the NICU for at least 3 days, the ability to speak Persian, not having anxiety and any mental or cognitive-sensory diseases (such as blindness and deafness) according to the patient, and not having any children with congenital abnormalities. The exclusion criteria were the absence of the mother for more than one session in the training program, failure to perform assigned tasks, withdrawal of the mother from the program, or the infant's death.

### Sample size

The sample size was estimated based on the data from a pilot study with 80% test power (β) and 5% error rate (α) and using Kopak's formula. To this end, 25 persons were assigned to each of the control and intervention groups with a 15% dropout rate.

### Instruments

The data in this study were collected using a demographic information questionnaire and the Spiritual WellBeing Scale (SWBS; Paloutzian and Ellison, 1991). The demographic information questionnaire recorded information such as the participants' education, occupation, history of chronic diseases, infant's gender, type of delivery, and history of previous children's admission to the NICU.

The Spiritual WellBeing Scale (SWBS) was developed by Paloutzian and Ellison (1982) to examine spiritual health. This 20-item scale measures two domains: religious health (10 items) and existential health (10 items). The odd items measure religious health and even items assess existential health. The items are scored on a 6-point Likert scale from strongly agree (6) to strongly disagree (1). The total score for each domain ranges from 10 to 60. Nine items are scored in reverse. Spiritual health is measured with a score range of 20–120 as follows: 20–40 (low spiritual health), 41–99 (moderate spiritual health), and 100–120 (high spiritual health). The reliability of the scale was confirmed with Cronbach's alpha of 0.82 (Paloutzian and Ellison, 1991; Paloutzian et al., 2021). The validity of the scale was confirmed for use in Iran by assessing its content validity and its reliability was confirmed with Cronbach's alpha value of 0.79 (Rezaie Shahsavarloo et al., 2016).

#### Data analysis

The collected data were analyzed using descriptive statistics (frequency, percentage, mean, and standard deviation) and inferential statistics. The results of the Kolmogorov-Smirnov test indicated that the data in this study followed a normal distribution. Thus, parametric tests were used. The independent samples t- test coping behaviors between the two groups before the intervention. Furthermore, the analysis of independent sample *t*-test was run to compare the two variables between the two groups after the intervention. The paired samples *t*-test was also used to compare the mean scores of the variables in each group. *P*-values < 0.05 were considered statistically significant.

After obtaining the necessary permits, the researcher attended the NICU and provided some instructions to the mothers about the objectives of the study and the procedure taken to conduct the intervention program. The mothers were also assured that their information would remain confidential and the findings would be published anonymously and would be made available to them upon their request. Moreover, written informed consent was obtained from the mothers who met the inclusion criteria.

Afterward, every other mother was randomly assigned to one of the control and intervention groups using a random number table, and the participants in the two groups completed the items in the demographic information questionnaire and the Spiritual WellBeing Scale.

The mothers in the intervention group were divided into groups of 4. Then they were asked to attend a room in the mental health clinic of the hospital on Saturdays every week for the intervention program. The sessions were conducted by a clinical psychologist, while the researcher (master's degree in neonatal intensive care nursing) was also present. The sessions were held from 9 to 11 in the morning because at this time the mothers were free after the doctor's visit and they had less stress and anxiety.

At the beginning of each training session, the researcher talked about the content of the session. Next, the clinical psychologist provided training. At the end of each session, meditation exercises were given to the mothers and they were asked to do the exercises at home.

The intervention involved teaching stretching exercise techniques, body scan meditation, meditation during daily activity, homework log form, 5-min listening exercise, 3-min breathing space, 20-min sitting meditation, discussion of mood, alternative thoughts and perspectives, breathing meditation, and mindfulness resources (Table 1).

**Table 1:** The content of the MBCT program.

**Sessions**	**Description**
1	Stating the meeting rules, defining mindfulness, spiritual health, coping strategies, stress and cognition, bodily techniques, and assigning homework
2	Reviewing the content of the previous session, receiving feedback on the exercises, the impact of our interpretation of events on our feelings, the impact of self-awareness and body awareness on life, seeing and listening exercises, homework
3	Reviewing the content of the previous session, receiving feedback on the exercises, defining cognitive errors and negative and positive thoughts, triangle expression, feeling, performance, understanding of cognitive errors, conscious walking techniques, conscious performance of a daily task, and homework
4	Reviewing the content of the previous session and receiving feedback, the impact of kindness, love, forgiveness on the mind, acceptance of thoughts instead of fighting them, real thoughts, automatic pilot, self-care, summarizing the instructions provided in all sessions, and homework

The mindfulness-based cognitive therapy intervention was conducted for 1 month. At the end of the program, the participants in the intervention group completed the items on the Spiritual WellBeing Scale.

To eliminate the possibility of sharing information by the mothers in the intervention group with the members of the control group, the intervention program was conducted in a separate space in the NICU. Besides, the mothers in the intervention group were asked not to talk with other mothers about the content of the training program. If a mother was absent from the sessions, an individual training session was held for her by the clinical psychologist. However, if a mother was absent from two sessions, she would be excluded from the study. Nevertheless, all the mothers in the intervention group completed the intervention program.

The mothers in the control group received routine NICU care and support, but they did not receive any other supportive intervention to overcome the anxiety and challenges caused by their infants' illness. One month later, the participants in the control group completed the Spiritual WellBeing Scale. To comply with ethical protocols, the intervention program was also conducted for the mothers in the control group at the end of the study.

## Findings

The participants in this study were 50 mothers of infants admitted to the NICUs of hospitals affiliated with Kerman University of Medical Sciences. The participants were placed into the mindfulness-based cognitive therapy group (*n* = 25) and the control group (*n* = 25). There was no statistically significant difference between the two groups in terms of the mother's demographic characteristics, occupation, education, type of delivery, history of abortion, history of chronic disease, and infant's gender, and the two groups were homogeneous in terms of demographic variables (*P* > 0.05) as shown in Table 2.

**Table 2:** The participants' demographic characteristics.

**Variable**	**Categories**	**Intervention group**		**Control group**		**Test results**
		**Frequency**	**%**	**Frequency**	**%**	
Education	Lower education	5	20	8	32	χ^2^ = 1.634
	Diploma	11	44	7	28	*P* = 0.442
	Higher education	9	36	10	40	
Occupation	Housewife	22	88	21	84	χ^2^ = 1.690
	Worker	0	0	1	4	*P* = 0.639
	Employee	2	8	1	4	
	Self-employed	1	4	2	8	
History of abortion	Yes	6	24	9	36	χ^2^ = 0.857
	No	19	76	16	64	*P* = 0.355
Neonatal gender	Female	7	28	5	20	χ^2^ = 0.439
	Male	18	72	20	80	*P* =0.508
Chronic diseases	Yes	2	8	1	4	χ^2^ = 1
	No	23	92	24	96	*P* = 1
Type of delivery	Natural	7	28	11	44	χ^2^ = 1.389
	C-section	18	72	14	56	*P* = 0.239

As shown in Table 3, the mean scores of spiritual health and its dimensions for the participants in the control group increased after the intervention compared to their scores before the intervention. However, the paired samples *t*-test showed no significant difference in the spiritual health scores of the participants in the control group before and after the intervention (*P* > 0.05). Moreover, the paired samples *t*-test showed a significant difference in the spiritual health scores of the participants in the intervention group before and after the intervention (*P* < 0.001), indicating that the short-term mindfulness-based cognitive therapy program improved spiritual health and its dimensions in the mothers of the infants admitted to the NICU after the intervention compared to before the intervention (Table 3).

**Table 3:** Comparing the research variables in the two groups.

**Variable**	**Groups**	**Pre-intervention scores**	**Post-intervention scores**	**Mean difference**	**Intragroup comparisons**
		**Mean** ±**SD**	**Mean** ±**SD**		
Spiritual health	Intervention	92.80 ± 2.14	104.04 ± 6.60	11.24 ± 6.50	*t* = −8.635
					*P* < 0.001
	Control	92.16 ± 16.35	92.56 ± 16.30	0.4 ± 1.25	*t* = −1.549
					*P* = 0.134
	Intergroup comparisons	t^**^= −0.876	F^***^= 96.063	t^**^= 8.169	
		*P* = 0.157	*P* < 0.001	*P* < 0.001	
			η^2^ = −0.671		
Religious health	Intervention	43.01 ± 7.23	49.28 ± 4.72	6.28 ± 3.52	t^*^= −8.896
					*P* < 0.001
	Control	43.16 ± 9.79	43.36 ± 9.78	0.20 ± 0.70	t^*^= −1.414
					*P* = 0.170
	Intergroup comparisons	t^**^= −0.066	F^***^= 91.632	t^**^= 8.444	
		*P* = 0.948	*P* < 0.001	*P* < 0.001	
			η^2^ = −0.661		
Existential health	Intervention	49.80 ± 6.33	54.76 ± 2.69	4.96 ± 4.62	t^*^= 5.364
					*P* < 0.001
	Control	49.01 ± 7.50	49.20 ± 7.48	0.20 ± 0.70	*t* = −1.414
					*P* < 0.001
	Intergroup comparisons	t^**^= −0.686	F^***^= 43.335	t^**^= 5.089	
		*P* = 0.407	*P* < 0.001	*P* < 0.001	
			η^2^ = 0.480		

^*^means that paired sample *t*-test was used.

^**^means that independent sample *t*-test was used.

^***^means that Fisher test was used.

The mean score of spiritual health in the intervention group was 92.80 ± 12.14 and that of the control group was 92.16 ± 16.35 before the intervention. The independent samples *t*-test showed no statistically significant difference between the mean scores of spiritual health for the participants in the intervention and control groups before the intervention (*P* = 0.876). Moreover, the mean score of spiritual health for the participants in the intervention group was 104.04 ± 6.60 and that of the control group was 92.56 ± 16.30 after the intervention, showing a significant intergroup difference (*p* < 0.001) (Table 3).

In addition, the mean score of religious health in the intervention group was 43.01 ± 7.23 and that of the control group was 43.16 ± 9.79 before the intervention. The independent samples *t*-test showed no statistically significant difference between the mean scores of religious health for the participants in the intervention and control groups before the intervention (*P* = 0.876). Additionally, the mean score of religious health for the participants in the intervention group was 49.28 ± 4.72 and that of the control group was 43.36 ± 9.8 after the intervention, showing a significant intergroup difference (*p* < 0.001) (Table 3).

The data also indicated that the mean score of existential health for the participants in the intervention group was 49.80 ± 6.33 and the corresponding value for the control group was 49.01 ± 7.50 before the intervention. The independent samples *t*-test showed no statistically significant difference between the mean scores of existential health for the participants in the two groups before the intervention (*P* = 0.686) (Table 3).

In addition, the mean score of existential health for the participants in the intervention group was 54.76 ± 2.69 and that of the control group was 49.20 ± 7.48 after the intervention, showing a significant intergroup difference (*p* < 0.001) (Table 3).

## Discussion

This study examined the impact of short-term mindfulness-based cognitive therapy intervention on the spiritual health of mothers of infants admitted to the NICU. The results indicated that the mean scores of spiritual health and its dimensions (religious and existential health) in the intervention group were significantly higher than the control group after the intervention, confirming the effectiveness of the mindfulness-based cognitive therapy intervention in promoting spiritual health and its dimensions in the mothers in the intervention group. In line with these findings, Park et al. (2020) examined the effect of MBCT on spiritual wellbeing, psychological distress, fear of cancer recurrence, fatigue, and quality of life in patients with non-metastatic breast cancer in Japan and reported that MBCT improved spiritual wellbeing and quality of life and reduced fear of disease recurrence and psychological stress in the patients. Although the intervention used in this study was similar to that used in the current study, the participants were women with cancer and had been involved in the disease and psychological distress caused by it for a longer period. Thus, they had different psychological conditions compared to the mothers of infants admitted to the NICU. Likewise, Labelle et al. (2015) showed that the mindfulness-based stress reduction (MBSR) program can improve spiritual health and post-traumatic growth (PTG) in breast cancer patients in the early stages of the disease and these variables were associated with lower levels of distress, improved quality of life, and greater wellbeing.

Contrary to the findings from the present study, Würtzen et al. (2015) examined the impact of an MBSR program for 6 and 12 months on distress, physical symptoms, spiritual health, and mindfulness in women with breast cancer and found that MBSR intervention improved the physical symptoms and mindfulness in the patients after 6 months. However, no significant difference was observed in the spiritual health of breast cancer patients during the follow-up phase (Würtzen et al., 2015). Perhaps the reasons for the conflicting findings reported in the two studies could be the longer duration of the patient's involvement with cancer, the completely different conditions of the mothers of the infants, the different spiritual and religious beliefs of the participants, the difference in the mindfulness interventions, and the length of the intervention program and the follow-up phase.

Consistent with the present study, Carmody et al. (2008) also showed that the mindfulness-based stress reduction program (MBSR) improves the characteristics of mindfulness and spirituality, and reduces reported medical symptoms and psychological distress due to the improvement of mindfulness and spiritual health in both sexes (Carmody et al., 2008). In this study, the MBSR program was effective in increasing mindfulness and spirituality, as reported in the present study. However, the participants were also different in the two studies. Moreover, the present study did not examine the mothers' medical and psychological symptoms. Furthermore, Aghili and Malek (2021) showed that mindfulness-based cognitive therapy (MBCT) was effective in improving spiritual health and reducing blood pressure in hemodialysis patients. Although the intervention used in this study was similar to the intervention conducted in the current study, the clients were suffering from physical pains caused by dialysis and special and chronic mental and emotional problems. Thus, their physical and psychological conditions were different compared to the mothers of infants admitted to the NICU.

However, a descriptive-analytical study by Dehghan et al. (2021) showed a significant and moderate relationship between hemodialysis patients' spiritual health and mindfulness. Furthermore, Mathad et al. (2019) reported a positive and significant relationship between mindfulness and spiritual wellbeing, self-compassion, and life satisfaction among undergraduate nursing students. Keng et al. (2011), in a review study aimed at investigating the impact of mindfulness-based stress reduction (MBSR) intervention on mental health among clinical and non-clinical populations and showed that MBSR improved the positive effects of spirituality. In this study, the participants and the type of mindfulness intervention were different from the current study, but the two studies reported similar findings (Keng et al., 2011). In addition, a meta-synthesis study by Benites et al. (2021) showed that cancer patients see spirituality as a factor in getting closer to God. Thus, promoting spirituality can be considered a solution to increase hope for the future, improve adaptation and coping, and create a good feeling in patients and their families (Benites et al., 2021). The findings of the studies reviewed above were in line with the data reported in the current study.

Concerning the effects of the mindfulness-based intervention on spiritual health, it is worth mentioning that given the religious and spiritual background of the Iranian community, most of the patients and their families consider many problems and diseases to be the will of God, and many parents use spirituality as a coping strategy in facing their children's critical conditions (Nematollahi et al., 2021). The mindfulness-based cognitive therapy intervention enhances individuals' spiritual health by changing their perspective on themselves, others, and the surrounding environment. It causes a person to refer to herself, and this reference to the self can be gradually deepened until the patient understands the feeling of wellbeing and feels that there is a superior force that by appealing to him can overcome problems and challenges (Aghili and Malek, 2021). As a result, it increases a person's ability to tolerate and effectively cope with psychological distress and reduces emotional tensions caused by stressful situations. The data in the present study showed that mindfulness-based cognitive therapy intervention improved the participants' spiritual health. Thus, this cognitive intervention can be used as a support measure to promote the spiritual health of mothers of infants admitted to the NICU.

## Limitations

This study was conducted with some limitations. First, the religion of all participants in the study was Islam, and there were no participants from other religions. Thus, the findings of this study may not reflect the spiritual needs of people following other religions that are less common in Iran. Second, during each MBCT session, the researcher also provided some recommendations and instructions to the mothers for the next session, and following these instructions was important for the intervention to be effective. However, the mothers showed different degrees of compliance with these instructions and recommendations, which the researcher had no control over. Another limitation of the study was the analysis of the impact of the intervention within 1 month after the intervention. Thus, future studies need to investigate the impact of the MBCT intervention over a longer period and with larger samples.

## Conclusion

The findings from the present study showed that the short-term mindfulness-based cognitive therapy intervention was effective in improving the spiritual and psychological health of mothers of infants admitted to the NICU. Thus, NICU nurses can use this mindfulness intervention to promote spiritual health, control psychological reactions, increase the sense of meaningfulness and meaning of life, and quality of life, and maintain the mental peace of mothers of infants admitted to the NICU. Improving the spiritual health of the mother can make it easier to deal with her mental crisis and as a result, improve the relationship between the mother and her infant and the spiritual wellbeing of the mother.

## Data Availability

The original contributions presented in the study are included in the article/supplementary material, further inquiries can be directed to the corresponding author.
